# Oscillatory Activity in the Infant Brain and the Representation of Small Numbers

**DOI:** 10.3389/fnsys.2016.00004

**Published:** 2016-02-08

**Authors:** Sumie Leung, Denis Mareschal, Renee Rowsell, David Simpson, Leon Iaria, Amanda Grbic, Jordy Kaufman

**Affiliations:** ^1^School of Health Sciences, Faculty of Health, Arts and Design, Swinburne University of TechnologyHawthorn, VIC, Australia; ^2^Centre for Brain and Cognitive Development, Department of Psychological Sciences, Birkbeck, University of LondonLondon, UK

**Keywords:** gamma-band activity, object permanence, small numbers, infancy, electroencephalogram, object processing

## Abstract

Gamma-band oscillatory activity (GBA) is an established neural signature of sustained occluded object representation in infants and adults. However, it is not yet known whether the magnitude of GBA in the infant brain reflects the quantity of occluded items held in memory. To examine this, we compared GBA of 6–8 month-old infants during occlusion periods after the representation of two objects vs. that of one object. We found that maintaining a representation of two objects during occlusion resulted in significantly greater GBA relative to maintaining a single object. Further, this enhancement was located in the right occipital region, which is consistent with previous object representation research in adults and infants. We conclude that enhanced GBA reflects neural processes underlying infants’ representation of small numbers.

## Introduction

How and whether infants appreciate that an out-of-sight object continues to exist remains a fundamental question in child psychology and developmental cognitive neuroscience. Based on Piaget’s original observations that infants under 9 months do not reach for hidden objects (Piaget, 1954), it was widely held that infants lack object permanence. However, recent studies measuring infants’ looking behavior suggested that infants as young as 2.5 months of age expect the continued existence of hidden objects (Wang et al., 2005), as they look longer at events that violate the permanence and solidity of objects than at events that do not have such violations. Electrophysiological and neuroimaging studies have revealed several possible underlying neural mechanisms for object retention in around 6-month-old infants (e.g., Csibra et al., 2000; Baird et al., 2002; Kaufman et al., 2003, 2005; Wilcox et al., 2005; Wilcox and Biondi, 2015).

One of these mechanisms is the gamma band synchronized neural activity (GBA), which underlies infants’ object tracking ability (Kaufman et al., 2003, 2005; Southgate et al., 2008), specifically, increased GBA at infants’ posterior temporal cortex was observed whenever an object was occluded (Kaufman et al., 2003). Importantly, this increase in GBA was not associated with the object’s disappearing state *per se*, but occurred most prominently when the manner of disappearance was consistent with the object’s continued existence (Kaufman et al., 2005). Such findings are similar to the enhanced GBA observed during a period that adults needed to hold an object representation in short-term memory (Tallon-Baudry et al., 1998). This enhancement has also been demonstrated to be specific to holding hidden objects in infants’ memory, as such increase was not seen with hidden faces (Southgate et al., 2008).

Although the importance of GBA for infants’ object processing has been established, it is not yet known whether the magnitude of this GBA relates to the amount of information infants maintain during object occlusion. Behavioral studies that examined infants’ object working memory capacity have been mainly divided into two lines of research: “how many” and “what”, with the former focusing on the number of individual objects that infants could track, and the latter focusing on the number of specific objects infants could identify (see Kibbe and Leslie, 2013). In the “how many” studies, infants as young as 4 months old could keep track of more than one hidden object at a time (Wynn, 1992; Mareschal and Johnson, 2003), and they had a upper limit of about three objects in the first year of life (Feigenson and Carey, 2003, 2005). These studies required infants to use spatiotemporal cues to individuate objects. They did not need to identify a distinct feature of the object (Xu and Carey, 1996; Leslie et al., 1998; Xu, 1999). In contrast, the “what” studies showed that infants of 6.5 months and younger could only hold the identity of one single item in short-term memory, (Káldy and Leslie, 2003, 2005; Ross-Sheehy et al., 2003), as this line of research required infants to recall featural information to individuate objects (Wilcox and Baillargeon, 1998; Wilcox, 1999; Wilcox and Schweinle, 2002; Wilcox et al., 2010).

The different upper limits in infants’ ability to retain the quantity vs. the identity of objects could be explained by how the brain processes different traits of an object differently, and the immaturity of these processes in infants. There are two routes for visual object processing: the dorsal route mainly processes spatial and temporal object information involved in guided action, such as location, whereas the ventral route mainly processes information that identifies an object (e.g., Ungerleider and Mishkin, 1982; Livingstone and Hubel, 1988; Milner and Goodale, 1995). While these routes are no longer thought to be as independent as they once were (see for example, Merigan and Maunsell, 1993; Puce et al., 1998; Humphreys and Jane Riddoch, 2003; Puce and Perret, 2003). Numerous developmental authors invoke the dual stream hypothesis as one of the most important heuristic frameworks for understanding early human infant-object interactions (e.g., Leslie et al., 1998; Xu et al., 1999; Atkinson, 2000; Johnson et al., 2001; Wilcox and Schweinle, 2002; Káldy and Sigala, 2004). Of note, is the finding that 4-month-old infants are capable of recalling the feature (via the ventral route), or the location (via the dorsal route) of an object separately, but unable to recall the combined feature and location information, suggesting that their ability to integrate information processed separately by the dorsal and ventral visual processing routes during occlusions is limited (Mareschal et al., 1999; Kaufman et al., 2003; Mareschal and Johnson, 2003; Mareschal and Bremner, 2006).

Infants’ attenuated GBA activity for hidden faces led researchers to believe that the GBA during occlusion does not reflect the ventral route of visual processing (Southgate et al., 2008). However, it has not been examined if the GBA observed in the previous occlusion studies (Kaufman et al., 2003, 2005; Southgate et al., 2008) underlies the activity of the dorsal route, which processes spatial temporal information that allows infants to individuate objects. The aim of the present study is to answer the question of whether the amount of GBA reflects the amount or number of items that become occluded. If so, this could indicate that the GBA observed in the previous occlusion studies reflects the processing of spatiotemporal information. As previous studies have shown an increase of brain activities in the alpha- and gamma-band when adults were asked to hold more items in their memory (Howard et al., 2003; Palva et al., 2011; Spitzer et al., 2014), we hypothesize that the GBA observed in infants’ object tracking would increase with the number of objects being occluded.

## Materials and Methods

### Participants

Twenty-eight full-term 6–8 month-olds (*M* = 212 days; 14 male, 14 female) participated in this experiment. An additional 13 infants were tested but were excluded from further analysis due to insufficient trial counts (fewer than 10 trials per condition) caused by fussiness or motion artifact. The study was approved by the Human Research Ethics Committee, Swinburne University of Technology, and written informed consent was obtained from the parents of all infant participants.

### Data Acquisition

Infants sat in a dimly-lit room on a parent’s lap, 60 cm from the stimulus monitor. EEG was recorded with Netstation 4.3.1. acquisition software, and a NA300 amplifier from a Hydrocel Geodesic Sensor Net comprised of 124 electrodes. Online, EEG data were sampled at 500 Hz and were referenced to the vertex electrode. Infants’ looking behavior was monitored and simultaneously video-recorded with the EEG data.

### Paradigm

**Figure 1:**
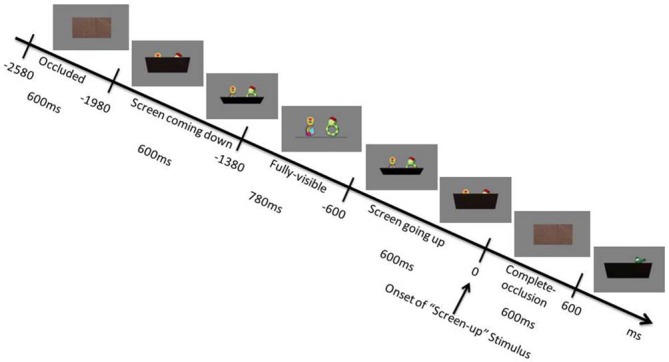
**A schematic presentation of the trial sequence**.

The experiment began with a stationary digital color photo of either two objects showing side by side, one object on the left side of the monitor, one object on the right side of the monitor or no object. The object(s) were fully visible for 780 ms (“fully-visible period”). It was followed by a gray screen moving upwards gradually until it covered the object entirely and was fully “up”, and this process took 600 ms. The objects remained completely occluded for 600 ms (“complete-occlusion period”). The gray screen then started to come down and revealed the next object(s), and the process took 600 ms (see Figure 1). An experimenter monitored the infants’ looking behavior would pause the experiment and played a movie to re-engage infants’ attention to the monitor before resuming the experiment. The conditions were collected pseudorandomly, with the 2-object and 1-object stimuli being presented no more than three times in a row, and the no-object stimulus never being presented twice in a row. The purpose of having a no-object stimulus was to introduce randomness to the paradigm, thus there were fewer no-object presentations. An average of 53.36 (range: 31–83) and 50.86 (range: 29–92) stimuli were presented for the 1-object and 2-object conditions, respectively, while the average number of presentations of the no-object stimulus was 32.14 (range: 7–60). A researcher monitored infants’ looking behavior via video link from another room, and whenever an infant looked away, would play a cartoon on the screen (with sound) to attempt to re-engage attention. The study was resumed when the infants looked at the screen again, and continued as long as the infants were happy.

### Data Analysis

EEG data were bandpass filtered (1–100 Hz, 12 dB/octave, 50 Hz notch). As we were interested in the GBA to the number of objects being occluded, we grouped the data into two stimulus conditions: 2-object and 1-object, and we analyzed the GBA during the period that the screen was fully up and stationary, and the objects were fully occluded (herein referred to as “complete-occlusion period”). For each of the stimulus conditions, EEG data were segmented from 1018 ms before the time when the screen was fully “up” (herein referred to as “screen-up”) to 982 ms post screen-up, and an independent component analysis (ICA) was applied to remove eye movement and blink artifacts for the whole segment. An automatic rejection was then applied, where segments with EEG amplitude variations larger than 200 μV between 182 ms pre screen-up to 818 ms post screen-up were rejected. Segments were rejected, if infants looked less than a total of 200 ms during the fully-visible period and less than a total of 300 ms during the complete-occlusion period. This resulted in an average of 29.65 (SD = 11.90) and 28.05 (SD = 15.10) segments for 1-object and 2-object conditions, respectively. There were at least 10 accepted segments for each of the conditions (1-object and 2-object) for each infant. In this paradigm, no baseline correction was used, because: (1) our two conditions are comparable and independent from each other, especially our expected effect is a tonic, rather than a phasic, modulation of GBA; and (2) there is not a period that is the same prior the occlusion period in the two conditions, as the periods prior to the screen contain either one or two objects visible respectively in the two conditions. We therefore used the 1-object condition as the “baseline” for the 2-object condition.

**Figure 2:**
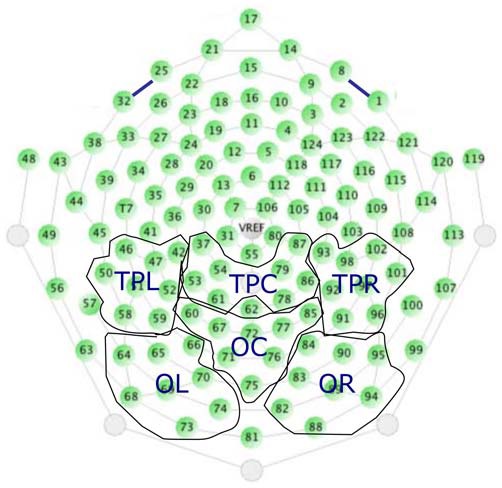
**The 48 posterior channels and their pre-defined groupings used for data analysis.** The six regions are: Temporal-Parietal-Left (TPL); Temporal-Parietal-Central (TPC); Temporal-Parietal-Right (TPR); Occipital-Left (OL); Occipital-Central (OC); Occipital-Right (OR).

Induced GBA was obtained by using a continuous wavelet transformation to the accepted segments of each electrode (Morlet wavelets with 21 frequency steps in the 30–50 Hz range). Average wavelet coefficients for each infant were calculated by taking the mean spectral amplitude (in μV) across segments during the complete-occlusion period, in two 300 ms bins (0–300 ms; 300–600 ms). Given that we previously found the object permanence GBA are located in the right posterior temporal cortex (Kaufman et al., 2003, 2005), we first grouped 48 posterior channels into six different regions: Temporal-Parietal-Left (TPL); Temporal-Parietal-Central (TPC); Temporal-Parietal-Right (TPR); Occipital-Left (OL); Occipital-Central (OC); Occipital-Right (OR); see Figure 2, then we calculated the mean gamma-band wavelet coefficients of eight electrodes for each of these regions.

### Statistical Analysis

To determine whether there was any effect due to the number of objects, one repeated measures analysis of variance (ANOVA) was employed where gamma-band wavelet coefficient during complete-occlusion period was the dependent variable, Condition (2-Object; 1-Object), Region (TPL; TPC; TPR; OL; OC; OR) and Latency (Early: 0–300 ms; Late 300–600 ms) were the independent variables. Greenhouse-Geisser correction was applied if the assumption of sphericity was violated. Where significant interactions were found, *post hoc* analysis were performed with Bonferroni correction for Type I error.

## Results

**Figure 3:**
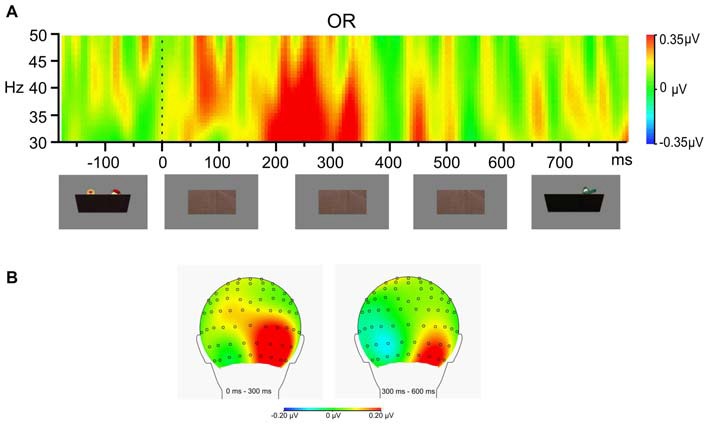
**Difference in gamma-band activity between 2-Objection condition and 1-Object condition during stimulus occlusion. (A)** Time frequency analysis of the average EEG of the eight electrodes in the pre-defined OR region. **(B)** Topographical maps of the gamma band (30–50 Hz) activity during the first half (0–300 ms) and the second half (300–600 ms) of the occlusion period.

The 2-Object condition generated more GBA than the 1-Object condition overall (*F*_(1,27)_ = 26.43, *p* < 0.001), and this interacted with Region (*F*_(5,135)_ = 152.24, *p* < 0.001). There was also a significant Region effect (*F*_(5,135)_ = 66.91, *p* < 0.001). Examining the significant interaction between Condition and Region, *post hoc* analyses for each of the six regions revealed that the 2-Object condition elicited more GBA than the 1-Object condition only at the Occipital Right region (*F*_(1,27)_ = 11.50, *corrected p* = 0.012), but no difference between the two conditions at any of the other five regions (see Figure 3).

## Discussion

The most meaningful finding of this study of young infants was that maintaining a representation of two objects during occlusion resulted in significantly greater GBA relative to maintaining a single object. Importantly, this enhancement was observed during the object occlusion period, in which there were no visible differences between the two conditions, thus demonstrating that these differences reflect distinct cognitive demands rather than perceptual processing. Similar to the enhanced GBA observed in adults when their working memory load increases (Howard et al., 2003; Palva et al., 2011), the current results support the hypothesis that the amount of GBA reflects the amount of perceptual history infants maintain after the objects were occluded.

The increase in GBA in the current study was in the right occipital region, which was more posterior than that reported in related earlier work (Kaufman et al., 2005), where GBA in the right temporal region during the occlusion period was higher than that during the disintegration period. However, taking together our current and previous results, the topographic distribution of the GBA during object occlusion in infants is similar to that in the left occipitotemporal area that Tallon-Baudry et al. (1998) observed in adults, in which subjects were told to keep an object in mind.

Interesting questions are raised however on the topographic differences between the current findings and those of Kaufman et al. (2003) who observed a marked gamma activity increase more specific to temporal cortex. This might be because GBA in that region is specific to holding any hidden object(s) in mind, regardless of how many objects, therefore any gamma change might become unobservable when we contrasted the two occluded conditions.

Another possibility which we think is more likely is that GBA in temporal cortex arises from the process of attempting to track the motion of an occluded object whereas the current study involved representing occluded stationary objects only. We think this explanation is more likely because of consistent evidence from both Southgate et al.’s (2008) work with infants and Tallon-Baudry et al.’s (1998) work with adults. Both of these studies involved the representation of stationary objects and resulted in similar topography to that of the infants described here. Future studies designed to differentiate the motion of occluded objects as opposed to occluded stationary objects will be needed to confirm this notion. Interestingly, the neural differences that we report between in the 1- and 2-object conditions are strikingly similar to what Southgate et al. (2008) reports when comparing activity during the occlusion of a single toy to the occlusion of a single face. Future studies are also needed to clarify what this fascinating similarity might represent.

As the GBA revealed here is generally consistent with prior work with occluded objects, it is worth reflecting on what this activity reveals about the neural processes underlying infant representation of small number. Our favored interpretation of this is that this type of brain activity underlies our early ability to represent small numbers (e.g., Wynn, 1992). However, we cannot at this point rule out the possibility that this activity is at least partially influenced by the total amount of visual input received prior to the occlusion period. For example, it is possible that occluding a single large object would results in the same pattern of activity as two smaller objects.

While additional research is necessary to definitively disentangle these possibilities, theoretical accounts of the role of GBA as well as behavioral studies with infants and adults suggest otherwise. For example, Cordes and Brannon (2008) specifically investigated size and number representations of young infants. Their results clearly showed that even when cues such as object size are available that infants spontaneously represent number. This work is consistent with both infant work (e.g., Feigenson and Carey, 2005) and adult work demonstrating that number representation often can take precedence over size representation (e.g., Gallivan et al., 2011). Moreover, it is important to note that in our two-object displays the objects were not contiguous. Given young infants use of contiguity to visually individuate objects (Kaufman and Needham, 2010), it is reasonable to assume that the brain activity reported here reflects individual object representation rather than total amount of visual input.

It is worth acknowledging the microsaccadic activity could present a potential confounding factor, as some research (e.g., Yuval-Greenberg et al., 2008) has suggested that this muscle-based activity can erroneously be measured as brain-based. However, we do not think this is an issue in the current study, because the reported differences occur when infants in the two conditions are viewing the identical scene (i.e., an occluding screen in the upright position). Thus, any GBA difference observed is best explained by the differences that define the two condition: number of objects prior to occlusion.

A number of important questions follow from this research; the most obvious being: how does GBA reflect larger numbers of occluded objects. Moreover, knowing that GBA can distinguish one from two hidden objects opens up opportunities for future research examining neural signatures for object individuation. In conjunction with the current research such studies should reveal a much richer picture of how neurodevelopment relates to cognitive change in preverbal infants.

## Author Contributions

SL, DM and JK prepared the manuscript; SL, RR, JK and AG did the analysis; JK, DM, DS, LI and SL designed the study; RR, AG and LI did the recruitment and testing.

## Funding

This research was supported under Australian Research Council’s Discovery Projects funding scheme (project number DP110101598). The Eric O. Baker Charitable Fund provided funding for the equipment used in this research. Finally, DM is partially funded by a Royal Society-Wolfson Research Merit award.

## Conflict of Interest Statement

The authors declare that the research was conducted in the absence of any commercial or financial relationships that could be construed as a potential conflict of interest.
